# Economical High–Low Temperature and Heading Rotation Test Method for the Evaluation and Optimization of the Temperature Control System for High-Precision Platform Inertial Navigation Systems

**DOI:** 10.3390/s18113967

**Published:** 2018-11-15

**Authors:** Qiang Yang, Rong Zhang, Haixia Li

**Affiliations:** Department of Precision Instrument, Tsinghua University, Beijing 100084, China; yangq16@mails.tsinghua.edu.cn

**Keywords:** inertial navigation system, temperature control system, evaluation method, optimization method, gimbal, platform, high–low temperature test, heading rotation

## Abstract

Inertial navigation systems (INSs) use the temperature control system to ensure the stability of the temperature of the inertial sensors for improving the navigation accuracy of the INSs. That is, the temperature control accuracy affects the performance of the INSs. Thus, the performance of temperature control systems must be evaluated before their application. However, nearly all high-precision INSs are large and heavy and require long-term testing under many different experimental conditions. As a result, conducting an outdoor navigation experiment, which involves high–low temperature and heading rotation tests, is time consuming, laborious, and costly for researchers. To address this issue, an economical high–low temperature and heading rotation test method for high-precision platform INSs is proposed, and an evaluation system based on this method is developed to evaluate the performance of the temperature control systems for high-precision platform INSs indoors. The evaluation system uses an acrylic chamber, exhaust fans, temperature sensors, and an air conditioner to simulate the environment temperature change. The outer gimbals of the platform INSs are utilized to simulate the heading rotation. The temperature control system of a high-precision platform INS is evaluated using the proposed evaluation method. The temperature difference of the gyros is obtained in the high–low temperature test, and the temperature fluctuation of the temperature control system is observed in the rotation test. These tests verify the effectiveness of the proposed evaluation method. Then, the corresponding optimization method for the temperature control system of this high-precision platform INS is put forward on the basis of the test results of the evaluation system. Experimental results show that the maximum temperature differences of the two gyros between high- and low-temperature tests are decreased from 1.51 °C to 0.50 °C, and the maximum temperature fluctuation value of the temperature control system is decreased from 0.81 °C to 0.27 °C after the proposed evaluation and optimization processes. Therefore, the proposed methods are cost effective and useful for evaluating and optimization of the temperature control system for INSs.

## 1. Introduction

Inertial navigation systems (INSs) can provide position, velocity, and attitude independently [1]. Thus, they are used extensively in land [2,3], marine [4,5], and aerospace navigation [6]. A direct and effective way to improve the navigation accuracy of an INS is to increase the accuracy of its inertial sensors. Unfortunately, the fluctuation in environment temperature [7] and the increase in temperature in running the navigation system lead to zero bias [8,9,10], scale factor errors [9,11] of the sensitive element, and deterioration of relevant electric circuits. These problems cause inertial sensor errors.

To compensate for the inertial errors caused by the fluctuation in the environment temperature [12], two kinds of methods have been proposed as follows. One is the passive method [10,13], which can include thermal compensation, thermal shunting of elements, and special structural design. Yang et al. [13] used tri-axial Micro-Electro-Mechanical System (MEMS) gyroscope outputs in different temperatures to calculate the error parameters of different temperature points for error compensation of gyroscope caused by temperature fluctuation. The other is the active method, which involves designing a temperature control system [14,15,16,17,18,19,20] to keep the temperature of the inertial sensors within a certain range while maintaining a high accuracy value. The former method requires a full understanding of all temperature effects. However, many temperature effects cannot be predicted effectively or modeled precisely [21]. Thus, the latter method, which entails adding a temperature control system for the INS, is often adopted. With regard to current studies, Giordano et al. [18] used cooling fans to control the temperature ultrasound obstacle detection system. Zhu et al. [19] utilized heaters to stabilize the temperature of the gyroscope. Cao et al. [20] used the thermoelectric coolers, fan and heater elements and temperature sensors to adjust the temperature of the sensor box. Li et al. [22] adopted fans, heater elements and fuzzy control algorithm to control the temperature of the INS. Xia et al. [23] adopted thermoelectric coolers and fuzzy-PID controllers to develop a temperature control system for silicon micro-gyroscope. Recently, Mironova et al. [24,25,26] used the thermoelectric cooler, fan and Lyapunov based control strategy for workpiece clamping system. The Lyapunov based control strategy guarantee the control of the nonlinear thermal system thermal systems and it has a potential use in INSs. Section 2 briefly introduces the temperature control system of the high-precision INS.

The control accuracy of the temperature control system affects that of the inertial sensors. The performance of the temperature control system should be evaluated before its application. However, a car test, a sea trial, or a space experiment is costly for researchers. Thus, a low-cost indoor evaluation system is urgently needed. On the one hand, the change in ambient temperature of INSs also changes the temperature fields outside the temperature control system. On the other hand, the heading rotation of the vehicle leads to relative position change of the gimbals, which changes the temperature fields inside the temperature control system. Both factors cause temperature fluctuation in the temperature control system. Therefore, the changes in heading rotation [27] and environment temperature are the main factors of the temperature fluctuation in the temperature control system.

At present, researchers in related fields propose evaluation methods for different INSs. Cao et al. [20] conducted a stability experiment inside a room without considering different environment temperature points. Li et al. [22] performed a temperature test by using a lamp to change the internal temperature of the INS. Xia et al. [23] tested the performance of the temperature control system in a temperature chamber that can change the external temperature of the inertial sensors. Levinson et al. [16] used a SMV temperature chamber to change the external temperature of a two-axis indexed Laser Gyro MK49 system. In summary, temperature tests have been adopted widely to test the performance of temperature control systems. However, the effect of the heading rotation is often neglected in the temperature tests. Temperature chambers to support the heading rotation and temperature change for the gyroscopes of low-precision, small-sized, and lightweight MEMS INSs are available [13]. Nevertheless, no evaluation system can support the high–low temperature and heading rotation of the high-precision, large-sized, and heavy platform INSs.

The evaluation system should contain a rotation functional unit (e.g., a turntable to rotate the INS [28,29,30,31]) and a temperature control functional unit (e.g., a high–low temperature chamber) to change the temperature of the environment. The weight of high-performance INSs often reaches 100–300 kg {e.g., MK39 (weight = 317.5 kg) [32], MK49 (weight = 317.5 kg) [33], AN/WSN-5L (weight = 172.7 kg) [34], NCS-1 (weight = 77.3 kg), and ESGN (weight = 405 kg) [35]}. To date, no indoor equipment can support high–low temperature and rotation test for the aforementioned systems. This study proposes an economical indoor evaluation method to evaluate the temperature control system for high-precision platform INSs with rotation gimbals for solving the above-mentioned issue.

On the basis of the test results of the evaluation system, the corresponding optimization method is proposed for the temperature control system of a high-precision platform INS. The temperature control system in this study is developed by Li et al. [22] in accordance with the special requirements of our previously developed high-precision platform INS. The current study mainly involves the evaluation and optimization of the temperature control system of this INS.

The remainder of the paper is organized as follows. The principle and structure of typical temperature control systems of high-precision INSs and the temperature control system of our INS are introduced in Section 2. The economical indoor evaluation method that contains high–low temperature test method (Section 3.1) and heading rotation test method (Section 3.2) for the temperature control systems of high-precision platform INSs is introduced in Section 3. The evaluation results of the temperature control system of a high-precision platform INS are provided in Section 4. The corresponding optimization method for the temperature control system is given in Section 5. The test results after optimization for the temperature control system are described in Section 6. Finally, the conclusions and future research directions are discussed in Section 7.

## 2. Temperature Control Systems of INSs

### 2.1. Typical Temperature Control System of INSs

**Figure 1:**
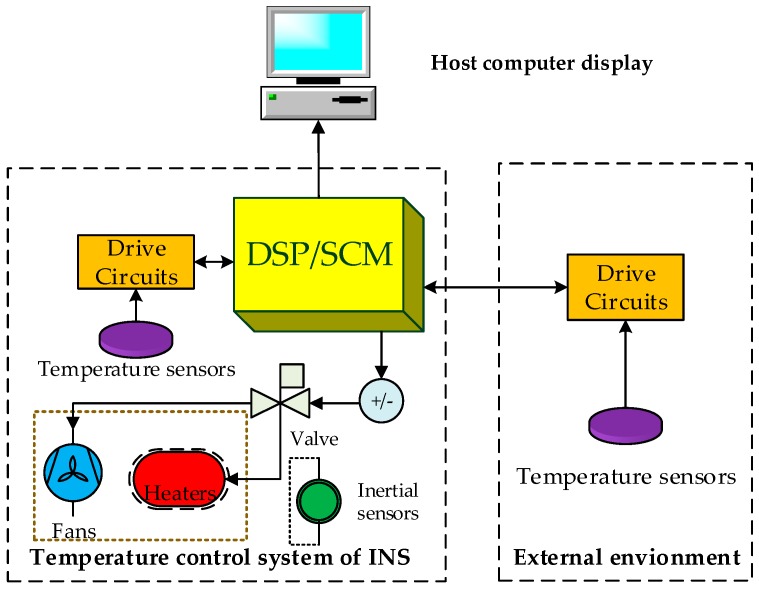
Platform of the temperature control system hardware.

The temperature control system is often utilized to ensure that the temperature of the inertial sensors is close to constant and is less affected by the environment temperature fluctuation for the INSs. That is, the temperature control system keeps the temperature of the inertial sensors within a certain range while maintaining a high accuracy value. A typical temperature control system uses heaters (fans) to heat (cool) the INS in accordance with the temperature measured by the sensors (Figure 1).

Most high-precision INSs adopt the method of incorporating a closed chamber with an approximately constant inner temperature. The temperature control system can adjust the internal temperature of the INSs automatically using heaters and fans depending on the difference of the current and target temperature. The heater is used to heat the internal temperature of the INS when the temperature is low or the temperature decreases quickly. The fan is used to cool the internal temperature when the temperature is high or the temperature increases rapidly. Then, the current temperature can be adjusted to the target temperature using the closed-loop temperature control method as [24]. Using the temperature control system, the internal temperature of the INS can be kept within a certain range to improve the accuracy of the inertial sensors. The temperature control system used here is developed by Li et al. [22]. The detailed principle of the temperature control can be found in reference [22]. The evaluation and optimization of the temperature control system are conducted by us.

### 2.2. Temperature Control System of Our INS

**Figure 2:**
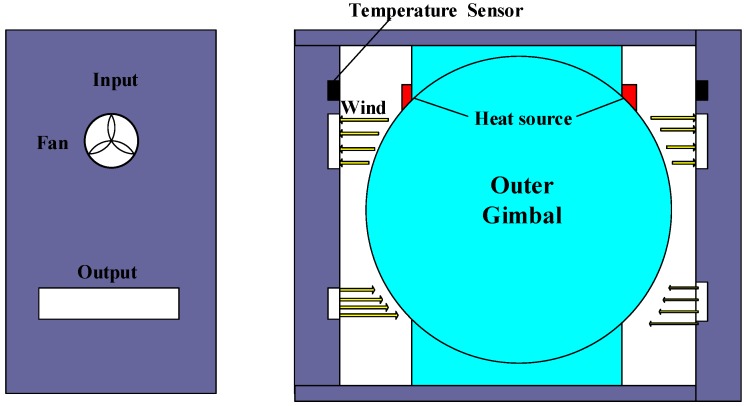
Structure of temperature control system of our high-precision platform INS.

The structure and working mechanism of the temperature control system of our high-precision platform INS are shown in Figure 2. The initial model of the temperature control system is developed by Li et al. [22] in accordance with the special requirements of this high-precision platform INS. Since this study mainly focuses on the design of a generally applicable evaluation system and structure optimization of INSs, we still use the algorithm in [22] for control algorithm. In the future, in order to further improve the control accuracy of temperature control system, we can use the PID [36], fuzzy-PID and Lyapunov based control strategies to compare the current control strategies of our INSs.

Figure 2 provides the physical structure schematic of the sealed temperature control system. The measuring temperature sensors used for the temperature control system are the TMP275 digital thermometers, which are installed on the inner walls of the chamber with a temperature resolution of 0.0625 °C. The inlet air temperature of the inner channel is measured by the four symmetrical distributions of the temperature sensors and the average value is selected as the observed temperature of the system. The closed structure can keep physical isolation of the measuring instrument with the external environment. Electric heaters are installed at the top of the apparatus to provide energy for heating the inner chamber, and their heat power is adjusted by the pulse width modulation (PWM) control method steps. The airflow between ambient environment and chamber is driven by the convection fans. The convection heat transfer intensity of the airflow channel is determined by the fan speed. In normal working conditions, the system will adjust the temperature of the chamber automatically by controlling the heaters and fans depending on the current and target temperatures.

## 3. Evaluation System of Temperature Control System

### 3.1. High–Low Temperature Test Method

**Figure 3:**
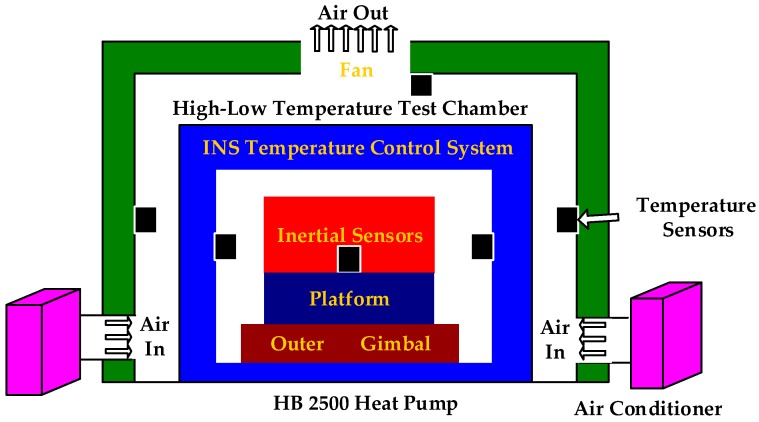
Configuration of the evaluation system.

To design a low-cost high–low temperature test system, the structure and equipment are designed as follows. The overall structure of the high–low temperature test chamber is made of acrylic plates consisting of a closed structure with an internal size of 150 cm × 120 cm × 90 cm (width × length × thickness). To simulate the change in environment temperature realistically, the high–low temperature test chamber is composed of acrylic plates, temperature sensors, and an air conditioner (Figure 3).

In Figure 3, the blue area represents the temperature control system of INS. The INS comprises two dual-axis high-precision gyros with a drift rate of 0.001°/h mounted on a platform. Beyond the blue region, the high–low temperature test chamber is constructed. Its main components comprise an HB 2500 air conditioner, an exhaust fan, temperature sensors, and a box made of thermal insulation foam. The total cost of the economical high–low temperature test system is approximately $1000. The green area represents the box made of thermal insulation foam. Three DS18B20 digital thermometers, which serve as the temperature sensors with a temperature resolution of 0.0625 °C, are distributed on the internal face of the box. The air enters from the air conditioning duct on the bottom and exits by the exhaust fan on the crown wall. The temperature sensor values are displayed and stored simultaneously with a real-time temperature acquisition system developed using Microsoft Visual C++ 6.0. Given that the temperatures of the temperature control system and gyros reflect those of the gimbals and inertial sensors, respectively, these values are used to evaluate the performance of the temperature control system.

Using the above mentioned temperature chamber, we can simulate the high and low environment temperatures of an actual outdoor navigation economically and accurately. Therefore, we need to achieve the heading rotation only for simulating the complete experimental conditions that affect the performance of the temperature control system. The following section describes the method of achieving heading rotation indoors.

### 3.2. Heading Rotation Test Method

#### 3.2.1. Derivation of the Principle of Heading Rotation

**Figure 4:**
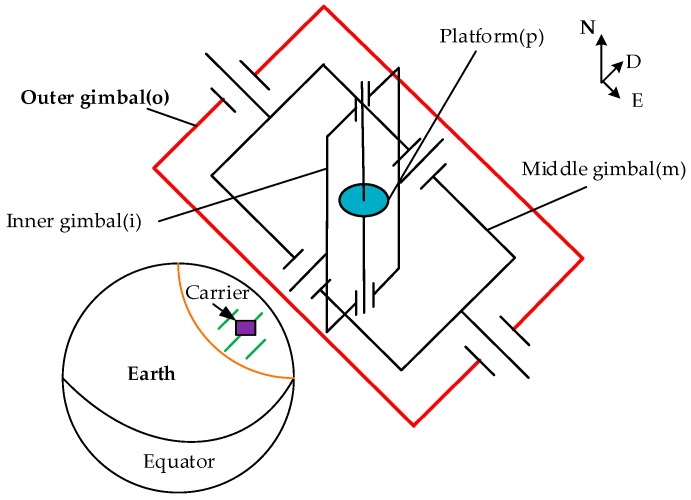
High-precision four-gimbal platform INS structure.

To simulate the heading rotation of the carrier, the mathematical relationship between the heading and the gimbal angles of the navigation system is deduced as follows. Here, the four-gimbal platform INS is taken as an example. As shown in Figure 4, the four-gimbal platform INS consists of the outer (color red), middle, inner, and platform gimbals. The motion of the carrier is isolated using servo control of the four gimbals.

**Figure 5:**
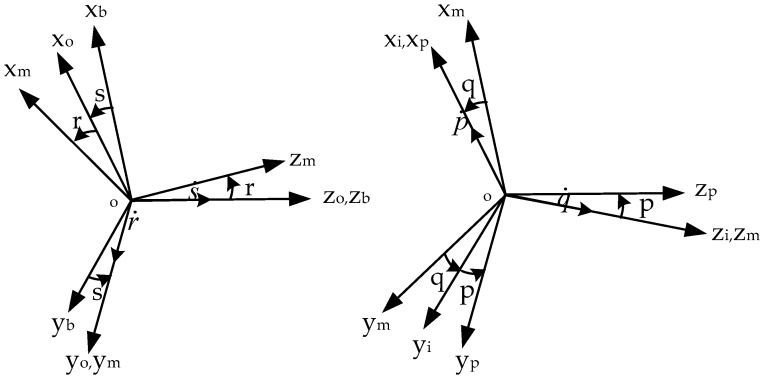
Four-gimbal platform coordinate system definition [37].

To establish the kinematics model of the four-gimbal platform INS, the coordinate systems connected to each part are introduced separately. Five coordinate systems are involved, namely, carrier body coordinate system (b-frame), outer gimbal coordinate system (o-frame), middle gimbal coordinate system (m-frame), inner gimbal coordinate system (i-frame), and platform coordinate system (p-frame). The geometric relationship among the coordinate systems is shown in Figure 5.

In Figure 5, p, q, r, and s represent the rotation angles of the platform, inner, middle, and outer gimbals, respectively; and $\dot{p}$, $\dot{q}$, $\dot{r}$, and $\dot{s}$ are their rotation rates. In this way, we specify the angular rate of the carrier motion as

(1) $$\omega_{b}={\left[\begin{array}{ccc} \omega_{bx} & \omega_{by} & \omega_{bz} \end{array}\right]}^{T},$$

where $\omega_{bx}$, $\omega_{by}$, and $\omega_{bz}$ are the angular rates components in the direction of three axes, respectively.

According to Figure 5, the angular rates of the coordinate systems have the following relations

(2) $$\left\{ \begin{array}{c} \omega_{o}={\left[\begin{array}{ccc} \omega_{ox} & \omega_{oy} & \omega_{oz} \end{array}\right]}^{T}={\left[\begin{array}{ccc} 0 & 0 & \dot{s} \end{array}\right]}^{T}+C_{b}^{o}{\left[\begin{array}{ccc} \omega_{bx} & \omega_{by} & \omega_{bz} \end{array}\right]}^{T}\\ \omega_{m}={\left[\begin{array}{ccc} \omega_{mx} & \omega_{my} & \omega_{mz} \end{array}\right]}^{T}={\left[\begin{array}{ccc} 0 & \dot{r} & 0 \end{array}\right]}^{T}+C_{o}^{m}{\left[\begin{array}{ccc} \omega_{ox} & \omega_{oy} & \omega_{oz} \end{array}\right]}^{T}\\ \omega_{i}={\left[\begin{array}{ccc} \omega_{ix} & \omega_{iy} & \omega_{iz} \end{array}\right]}^{T}={\left[\begin{array}{ccc} 0 & 0 & \dot{q} \end{array}\right]}^{T}+C_{m}^{i}{\left[\begin{array}{ccc} \omega_{mx} & \omega_{my} & \omega_{mz} \end{array}\right]}^{T}\\ \omega_{p}={\left[\begin{array}{ccc} \omega_{px} & \omega_{py} & \omega_{pz} \end{array}\right]}^{T}={\left[\begin{array}{ccc} \dot{p} & 0 & 0 \end{array}\right]}^{T}+C_{i}^{p}{\left[\begin{array}{ccc} \omega_{ix} & \omega_{iy} & \omega_{iz} \end{array}\right]}^{T} \end{array},\right.$$

where $\omega_{ij}$ is the angular rate, the subscript i represents the coordinate systems fixed with each frame, and j denotes the corresponding axis direction. The transformation matrixes among the coordinate systems are

(3) $$\{\begin{array}{cc} C_{b}^{o}=\left[\begin{array}{ccc} \cos s & \sin s & 0\\ -\sin s & \cos s & 0\\ 0 & 0 & 1 \end{array}\right]， & C_{o}^{m}=\left[\begin{array}{ccc} \cos r & 0 & -\sin r\\ 0 & 1 & 0\\ \sin r & 0 & \cos r \end{array}\right]\\ C_{m}^{i}=\left[\begin{array}{ccc} \cos q & \sin q & 0\\ -\sin q & \cos q & 0\\ 0 & 0 & 1 \end{array}\right]， & C_{i}^{p}=\left[\begin{array}{ccc} 1 & 0 & 0\\ 0 & \cos p & \sin p\\ 0 & -\sin p & \cos p \end{array}\right] \end{array}.$$

The transformation matrix between the carrier and platform coordinate systems is denoted as $C_{b}^{p}$. Thus,

(4) $$C_{b}^{p}=C_{i}^{p}C_{m}^{i}C_{o}^{m}C_{b}^{o}.$$

From Equations (1)–(4), the mathematical relationship among the angular motion of the platform, the gimbals, and the carrier are obtained as follows:

(5) $$\omega_{p}=C_{b}^{p}\omega_{b}+C{\left[\begin{array}{cccc} \dot{p} & \dot{q} & \dot{r} & \dot{s} \end{array}\right]}^{T},$$

where $\omega_{p}$ is the angular rate of the platform;

(6) $$C=\left[\begin{array}{cccc} 1 & 0 & \sin q & -\cos q\sin r\\ 0 & \sin p & \cos p\cos q & \cos p\sin q\sin r+\sin p\cos r\\ 0 & \cos p & -\sin p\cos q & -\sin p\sin q\sin r+\cos p\mathrm{cosr} \end{array}\right],$$

(7) $$C_{b}^{p}=\left[\begin{array}{ccc} \cos q\cos r\cos s-\sin s\sin q & \cos q\cos r\sin s+\sin q\cos s & -\sin r\cos q\\ \begin{array}{l} \cos s\left(-\cos p\sin q\cos r+\sin p\sin r\right)\\ -\sin s\cos p\cos q \end{array} & \begin{array}{l} \sin s\left(-\cos p\sin q\cos r+\sin p\sin r\right)\\ +\cos s\cos p\cos q \end{array} & \begin{array}{l} \cos p\sin q\sin r\\ +\sin p\cos r \end{array}\\ \begin{array}{l} \cos s\left(\sin p\sin q\cos r+\cos p\sin r\right)\\ +\sin s\sin p\cos q \end{array} & \begin{array}{l} \sin s\left(\sin p\sin q\cos r+\cos p\sin r\right)\\ -\cos s\sin p\cos q \end{array} & \begin{array}{l} -\sin p\sin q\sin r\\ +\cos p\cos r \end{array} \end{array}\right].$$

Our aim is to simulate the heading rotation, which is defined as the angle at which the carrier rotates counterclockwise around the z axis in the horizontal plane. Thus, we only need to discuss the angular rate vector component along the z axis. From Equation (5), we obtain

(8) $$\omega_{pz}={\left(C_{b}^{p}\right)}_{3i\left\{i=1,2,3\right\}}{\left[\begin{array}{ccc} \omega_{bx} & \omega_{by} & \omega_{bz} \end{array}\right]}^{T}+C_{3j\left\{j=1,2,3,4\right\}}{\left[\begin{array}{cccc} \dot{p} & \dot{q} & \dot{r} & \dot{s} \end{array}\right]}^{T}.$$

Given that high-performance inertial sensors are often used for the platform INS, the physical platform changes caused by gyro drift are neglected. We obtain

(9) $$\omega_{p}={\left[\begin{array}{ccc} \omega_{px} & \omega_{py} & \omega_{pz} \end{array}\right]}^{T}={\left[\begin{array}{ccc} 0 & 0 & 0 \end{array}\right]}^{T}.$$

To simplify Equation (8), we set the platform and inner gimbals to certain positions. The test can be conducted in a static horizontal base. Thus, p, q and $\dot{p}$, $\dot{q}$ are set to 0. Then, Equations (6) and (7) can be simplified as

(10) $$C=\left[\begin{array}{cccc} 1 & 0 & 0 & -\sin r\\ 0 & 0 & 1 & 0\\ 0 & 1 & 0 & \mathrm{cosr} \end{array}\right],$$

(11) $$C_{b}^{p}=\left[\begin{array}{ccc} \cos r\cos s & \cos r\sin s & -\sin r\\ -\sin s & \cos s & 0\\ \cos s\sin r & \sin s\sin r & \cos r \end{array}\right].$$

Substituting Equations (9)–(11) into Equation (8) yields

(12) $$\omega_{bx}\cos s\sin r+\omega_{by}\sin s\sin r+\omega_{bz}\cos r+\dot{s}\mathrm{cosr}=0.$$

Given that the INS is placed in a horizontal plane, the roll and pitch angles of the carrier remain unchanged. Thus, we can obtain

(13) $$\omega_{b}={\left[\begin{array}{ccc} \omega_{bx} & \omega_{by} & \omega_{bz} \end{array}\right]}^{T}={\left[\begin{array}{ccc} 0 & 0 & \omega_{bz} \end{array}\right]}^{T}.$$

Then, Equation (12) can be decreased into

(14) $$\omega_{bz}\cos r+\dot{s}\mathrm{cosr}=0,$$

where r is the relative rotation of the middle gimbal and is related to the local latitude of the carrier and the pitch value. It is a stationary base; thus, cosr is a fixed and a nonzero value. $\omega_{bz}$ is the angular rate of the vehicle heading in the static horizontal plane. Equation (14) can be written as the following final form:

(15) $$\dot{\psi }=\omega_{bz}=-\dot{s},$$

where $\dot{\psi }$ is the angular rate of the heading.

In accordance with Equation (16), the heading rotation of the INS can be simulated by rotating the outer gimbal.

#### 3.2.2. Outer Gimbal Rotation Test Procedure

Before the heading rotation test, we need to consider whether there is singularity in the whole rotation process. The singular points can cause unpredictable consequences of the system.

In the outer gimbal rotation test, firstly, p and q are set equal to 0 and unchanged, thus, $C_{m}^{i}$ and $C_{i}^{p}$ are identity matrices; Secondly, r is the relative rotation of the middle gimbal and is determined by the local latitude of the carrier and the pitch value. It is a stationary base indoors, thus, cosr is a fixed and nonzero value, except for the situation that systems are placed at the south pole and north pole points of earth and the pitch value is 90°; Finally, neither s nor the matrix containing s does not appear on the denominators in mathematical relations. Thus, there will be no singularities. The heading rotation of the INS can be simulated by rotating the outer gimbal with angle range 0~360°.

A rotation test can be initially conducted to identify the maximum temperature fluctuation regions for testing the performance of the temperature control system. The heading angle can be expressed as:

(16) ψ(t)=ψ(0)+k×t,

where ψ(t) is the heading at time t, ψ(0) is the heading angle at the initial time, and k is the heading angle rotation rate.

After the maximum temperature fluctuation point at heading angle $\psi_{c}$ is obtained, the periodic heading rotation test can be conducted. Then, ψ(t) can be expressed as

(17) $$\psi \left(t\right)=\psi_{c}+A\times \sin\left(2\pi f_{h}\times t\right),$$

where $f_{h}$ is the frequency of heading, $\psi_{c}$ is the mean value of the harmonic oscillation, and A is the swing amplitude value.

Using the continuous and periodic heading rotation test, the performance of the temperature control system can be evaluated quickly.

## 4. Evaluation Process for a High-Precision Platform INS

**Figure 6:**
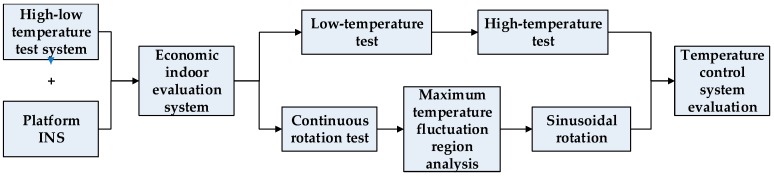
Flowchart of the evaluation method.

The proposed system can simulate the heading (azimuth) changes of the carrier and the environment temperature changes of the INSs. Figure 6 shows a typical evaluation process, which is conducted in the subsequent sections. The high–low temperature test system and the gimbals of the platform are combined to build the high–low temperature and heading rotation test system. The high–low temperature test is done to evaluate the temperature difference of the gyros in different environment temperatures. The continuous rotation test is adopted to identify the large temperature fluctuation field of the temperature control system, and the sinusoidal rotation test is performed to analyze this large temperature fluctuation field further. The temperature data are recorded in the tests, and the performance of the temperature control system can then be evaluated. In the following section, practical tests are conducted to verify the proposed method.

Heading rotation and high–low temperature tests are conducted to verify the performance of the proposed economical test method. The INS comprises two dual-axis gyros with low drift rates mounted on a four-gimbal platform. The high–low temperature test chamber is produced in accordance with Figure 3. The angle of the outer gimbal and temperatures of the inertial sensors, temperature control system and environment are retained. The performance of the temperature control system is evaluated using the test results. On the basis of the test results, the temperature control system can be improved and its performance can be re-verified.

### 4.1. Long-Term High–Low Temperature Test

The high–low temperature test is performed to simulate the change in the external environment temperature. The environment temperature changes slowly in practical use in a short time. In this case, the temperature fluctuation of temperature control system is very small. Thus, in the high–low temperature test, only the temperature fluctuation of the inertial sensors is calculated. In the high and low temperature tests, the environment temperatures are set to 29 °C and 19 °C, respectively. The fluctuations of the two gyroscopes are retained after subtracting a constant value at high and low temperatures in 24 h.

**Figure 7:**
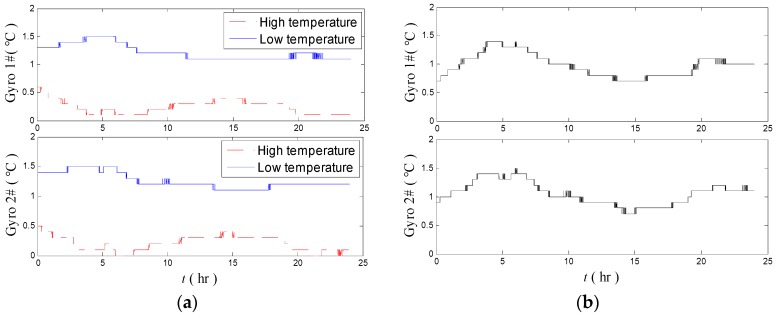
Temperature fluctuation of the gyros in the high–low temperature tests (**a**) and the temperature fluctuation difference of the gyros under high and low temperature tests (**b**).

Figure 7a shows the temperature fluctuations of the two gyros in the high–low temperature tests in 24 h. Figure 7b indicates the temperature calculated difference of the same gyros between the high- and low-temperature tests. The average temperature differences of both gyros are at 1.0 °C level, and the highest temperature difference reaches 1.51 °C. These findings imply that the temperature control system of the platform INS should be improved further.

### 4.2. Short-Term Heading Rotation Test

The heading rotation test is realized by rotating the outer gimbal. The rotation test of outer gimbal will directly change the internal temperature field of the temperature control system, which changes the temperature of the temperature control system quickly. However, the former change slightly affects the internal isolated gyroscopes in a short time. Accordingly, only the temperature fluctuation of the temperature control system is evaluated in the heading rotation test.

To determine the maximum temperature fluctuation region quickly, we find a relatively wide range of excessive temperature fluctuations, firstly, by rotating the outer gimbal uniformly. In this part, we propose a fast rotation method that utilizes the principle of four-equal-division of cycle and symmetry of the structure. The rotation process of the outer gimbal is given in Table 1.

**Figure 8:**
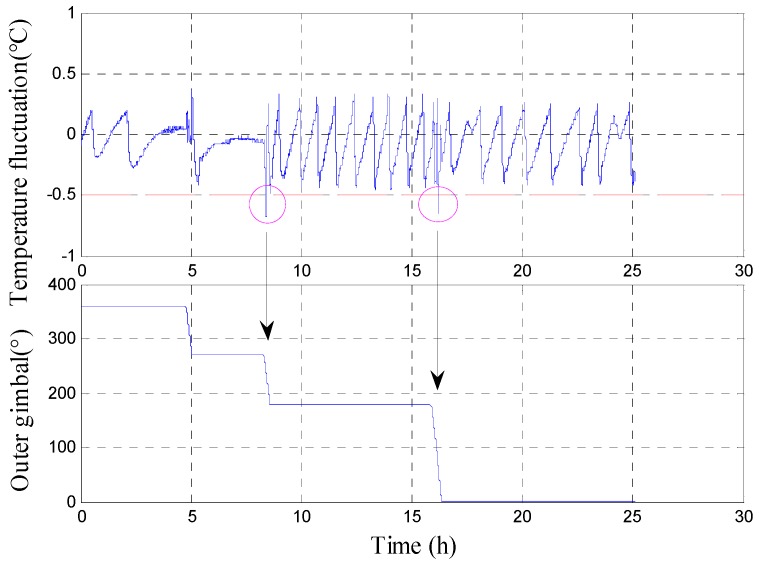
Temperature fluctuation of the temperature control system.

As shown in Table 1, the gimbal first rotates from 360° to 270° with a uniform rotation rate of 0.1°/s. The gimbal pauses at 270° for several minutes to decrease the effect of previous rotation to the next one. Then, the gimbal rotates from 270° to 180°. A large temperature fluctuation is observed. Thereafter, the outer gimbal rotates from 180° to 0° directly because the internal structure of the INS is approximately symmetric. The second region with excessive temperature fluctuation is observed as expected (Figure 8).

**Figure 9:**
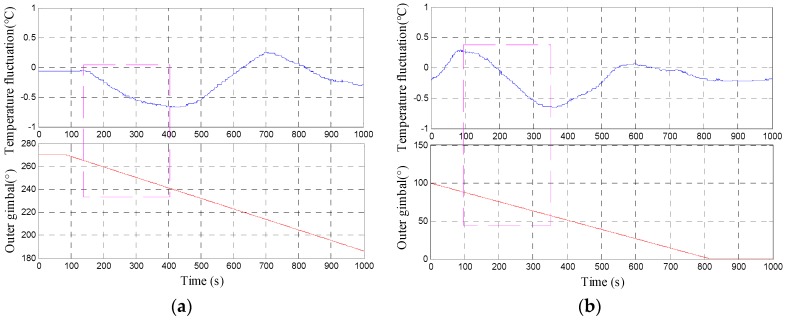
Temperature fluctuation of the temperature control system in the gimbal angle ranges of (**a**) 270–246° and (**b**) 92–54°.

Figure 8 shows the temperature fluctuation curve of the temperature control system, which is obtained by rotating the outer gimbal uniformly in the entire cycle. As shown in the figure, two regions have excessive temperature fluctuation. The symmetry of the two regions can be observed clearly in the partially enlarged Figure 9a,b, which indicates that the entire structure of the INS is symmetric. The maximum temperature fluctuation reaches 0.68 °C.

After the two regions with excessive temperature fluctuation are obtained, we make the outer gimbal rotate sinusoidally to simulate the heading change of the ship in actual navigation. To further analyze the fluctuation caused by the actual heading rotation, some outer gimbal rotations with sinusoidal period are carried out near the two regions with excessive temperature fluctuation. The rotation process and relevant parameters of two platform INSs are recorded in Table 2.

Table 2 lists the temperature fluctuation values of the two regions with excessive temperature fluctuation in different heading rotation processes, in which the maximum temperature fluctuation and its corresponding mean angle of the heading rotation test are in bold. Regions of around 64.5° with excessive temperature fluctuation and are symmetric in structure are found. The maximum temperature fluctuation point reaches 0.8125 °C. The temperature fluctuations in other regions are less than 0.4500 °C.

**Figure 10:**
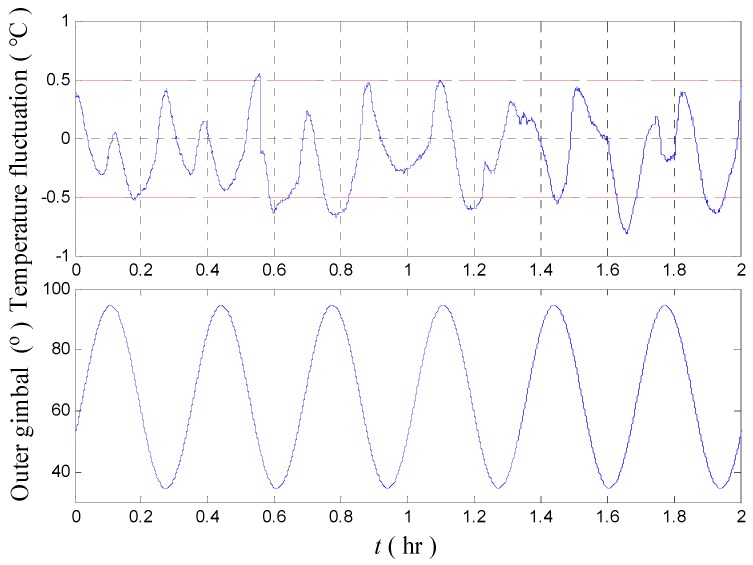
Temperature fluctuation of the temperature control system.

We take the maximum temperature fluctuation of INS 1# as an example. The mean value, rotation amplitude, and rotation period are 64.5°, 30°, and 1200 s, respectively. Given the structural symmetry of the two regions, only the experimental result conducted in one region (92–54°) is presented in Figure 10.

Figure 10 shows that excessive temperature fluctuations are again observed when the heading is set as a sinusoidal rotation. The test result is in line with the previous uniform rotation test. The maximum temperature fluctuation point reaches 0.8125 °C.

The temperature control system of the INS should be further improved on the basis of the results of the high–low temperature test (Section 4.1) and rotation test (Section 4.2). In the following part, the temperature control system of the high-precision platform INS is optimized using the evaluation results above.

## 5. Optimization of the Temperature Control System of the INS above

### 5.1. Analysis for Excessive Temperature Fluctuation

The reason for the large temperature differences of the gyros between the high- and low-temperature tests is analyzed as follows. The internal structure of the INS is shown in Figure 2. The temperature of the outlet of the heater reaches 46 *°*C when the ambient temperature is near 20 *°*C. The finding indicates that the temperature control system heats the INS when the ambient temperature is low. In this case, the hot wind will blow directly at the outer gimbal of the INS in the outlet of heater elements and suck at the gimbal in the inlet. Accordingly, the gimbal is heated directly and the temperatures of the gyros in the gimbal are increased to a high value (Figure 7). When the ambient temperature is near 28 °C, the heaters stop working. Moreover, the cool wind will blow and suck directly at the gimbal. Thus, the gimbal is cooled directly and the temperatures of the gyros are decreased to a low value. That is, the temperatures of the gyros will be increased in low ambient temperature and decreased in high ambient temperature. As a result, the temperature control system exhibits excessive temperature fluctuation (Figure 10).

The reason for the large fluctuation of the temperature control system in the heading rotation is analyzed as follows. The heat sources are close to temperature sensors when the gimbal rotates to 60–70 ° and 240–250°, as shown in Figure 8 and Figure 9. When the outer gimbal rotates to the position where the temperature sensors are close to the heat source, the value given by the temperature sensors increases quickly. Unfortunately, the values given by the temperature sensors are used to provide temperature reference for the temperature control system. Accordingly, the temperature control system will cool the air between the temperature control system and outer gimbal. If the outer gimbal rotates continually, then the temperature sensors move far from the heat sources. At the same time, the temperature of the air has been cooled by the temperature control system. Then, the temperature is decreased quickly (Figure 10). The fluctuation environment temperature is within 1.0 °C, but the temperature control error of the temperature control system reaches 0.6 °C.

### 5.2. Optimization of the Temperature Control System

**Figure 11:**
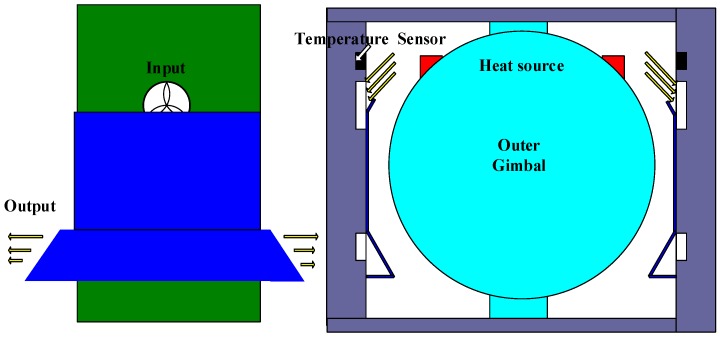
Optimization of the temperature control system.

To overcome the two shortages mentioned above, the structure between the temperature control system and the outer gimbal is optimized as shown in Figure 11. In the outlet, the copper plate is utilized to prevent the air from blowing directly at the outer gimbal, and the wind blows at the lower left and right sides of the space between the temperature control system and the outer gimbal. Thus, the temperature of gimbal is less affected by the heaters. In the inlet, the same copper plate is applied to accelerate air circulation by decreasing the inlet area and using a slant structure. In this way, the temperature sensors are less affected by the heat sources. Thus, the practical temperature of the air between the temperature control system and the gimbals is measured by the temperature sensors and adjusted by the temperature control system. The structure of the temperature control system is optimized by us. The specific control algorithm which can be found in reference [22] is not modified in this study.

The optimized temperature control system has three advantages compared with the original one. First, when the temperature of the heater is high, the air from outlet of the heater does not blow directly to the outer gimbal. This condition causes uniform air temperature distribution between the doors of the temperature control system and the outer gimbal. Second, when the heat element stops working and outlet air temperature is low, the wind from the outlet does not flow at the outer gimbal directly, which also increases the temperature of the gyros slightly compared with the original test. Then, the temperature of gyros decreases in the high-temperature test. Third, the temperature fluctuation caused by the gimbal rotation would be reduced as the temperature given by the temperature sensor less affected by the heat source. The optimization structure with the down air duct also protects the circuit of the temperature control system by preventing the hot gas from blowing directly to the circuit box.

After optimization, the practical temperature of the air between the temperature control system and the gimbals is measured by the temperature sensors more accurately. The temperature difference rate is also calculated using the temperature difference. Then both temperature difference and temperature difference rate are used to adjust the heating powers of the heaters and speeds of the fans according to the control strategy given in reference [22]. According to the temperature difference, temperature difference rate and control strategy, the air between the temperature control system and the gimbals will be heated or cooled using a better mathematical model of temperature field. Then the temperature of the air between the temperature control system and gimbals can easily be kept within a certain range.

## 6. Experimental Results after Evaluation System and Optimization

### 6.1. Temperature of Inertial Sensors

After optimization, the high–low temperature and rotation tests are conducted for the second time to verify the effectiveness of the proposed evaluation and optimization methods.

**Figure 12:**
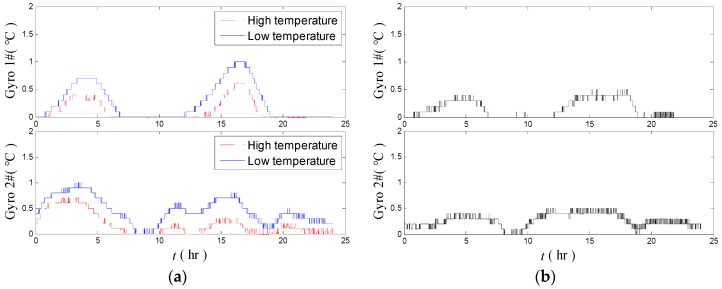
Temperature fluctuation of the gyros in the high–low temperature tests (**a**) and the temperature fluctuation difference of the gyros under high and low temperature tests (**b**) after the evaluation and optimization proposed methods.

As shown in Figure 12a, the temperature difference of the gyros in high- and low-temperature environments is decreased compared with that before optimization (Figure 7). The temperature fluctuations of gyros are below 1.0 °C in high- and low-temperatures. Figure 12b shows that the differences of temperature fluctuation of the two gyros under high and low temperatures are decreased to below 0.5 °C.

### 6.2. Temperature of Temperature Control System

Although two excessive temperature fluctuation fields exist, only the periodic rotation test conducted in the outer gimbal angle range of 94.5–34.5° is presented here in consideration of the symmetric structure of the INS.

**Figure 13:**
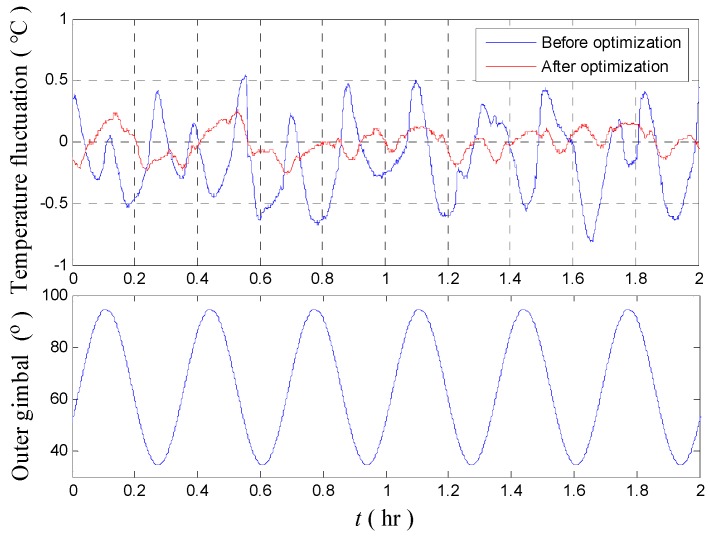
Temperature fluctuation of temperature control system before and after optimization in normal temperature.

Figure 13 shows that the amplitude of the fluctuation is decreased from 0.81 °C to 0.27 °C. This result confirms the improvement of the temperature control system after optimization. Table 3 presents the test results before and after optimization of the temperature control system of INS.

Table 3 lists the temperature fluctuation results of the gyros and temperature control system before and after optimization, in which the maximum fluctuation differences of gyros and maximum fluctuation of temperature control system are in bold. As shown in the table, all the experimental results of the performance parameters of the temperature control system have improved. Similar to what we have analyzed before, we focus on the most influential parameters of INSs, namely, the maximum fluctuation differences of the gyroscopes in high–low temperature test and the standard deviation temperature fluctuation of the temperature control system in heading rotation test. The maximum fluctuation differences of the two gyroscopes are drastically decreased from 1.5 °C and 1.4 °C to 0.5 °C. The standard deviation temperature fluctuation of the temperature control system is drastically decreased from −0.8125 °C to −0.2656 °C.

**Figure 14:**
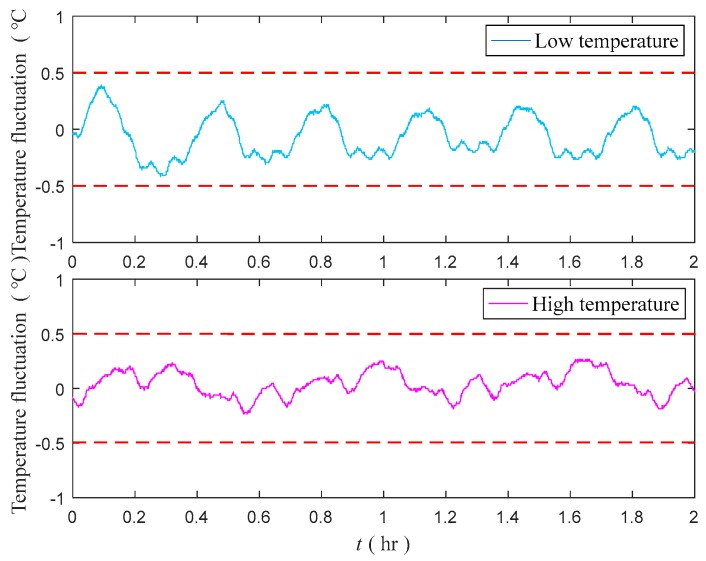
Temperature fluctuation of temperature control system after optimization in high and low temperatures.

To ensure the validity of our evaluation and optimization methods, we perform the heading rotation tests for the temperature control system at high and low environment temperatures. Figure 14 shows that the maximum temperature fluctuation value is less than 0.4500 °C.

The test results indicate that the economical high–low temperature test system can be utilized to determine the temperature difference of the gyros in dissimilar environment temperatures. The high–low temperature test shows that the temperature differences of the same gyro reach 1.50 °C. Meanwhile, the temperature fluctuation of the temperature control system caused by the heading rotation can be evaluated using the heading rotation test. The maximum temperature fluctuation region can be found first using the continuous rotation test and then analyzed by the periodic rotation test. The rotation test reveals that the temperature fluctuation reaches 0.81 °C. The test conclusions provide useful guidance for optimizing of the temperature control system. After optimization, the temperature differences of both gyros between high- and low-temperature tests are decreased from 1.51 °C to 0.50 °C. Moreover, the temperature fluctuation of the temperature control system is decreased from 0.81 °C to 0.27 °C. With the aid of the proposed economical high–low temperature and heading rotation test method, the performance of the temperature control system is evaluated. The performance of the temperature control system is improved using the proposed optimization method. As a result, the gyros have higher accuracy.

## 7. Conclusions

High-precision INS equipment is usually large and heavy. Thus, conducting high–low temperature and heading rotation tests is time consuming, laborious, and costly for researchers. To overcome this issue, we propose an economical high–low temperature and heading rotation test method to evaluate the temperature control system for high-precision platform INSs. The proposed high–low temperature system comprises a convenient and low-cost test chamber system and is utilized to simulate the environment temperature change. Then, the relationship between the heading and gimbal angles of the platform INS is deduced, and the outer gimbal is selected to simulate the heading rotation. With the high–low temperature system and the outer gimbal, a typical high–low temperature and heading rotation test process is also presented.

The proposed evaluation method is verified by high–low temperature and heading rotation tests. The obvious temperature difference of the gyros is found through the high–low temperature test. The maximum temperature fluctuation fields are also observed through the heading rotation test. The evaluation test results are useful for optimizing the temperature control system. The improvement of the temperature control system by optimization method is further re-confirmed by the proposed evaluation method. With the aid of the proposed evaluation and optimization methods, the temperature fluctuations of the temperature control system and gyros of the INS are decreased evidently. In the future, researchers can develop their own economical high–low temperature and heading rotation test system for their high-precision platform INSs using the evaluation method proposed in this study.

## Figures and Tables

**Table 1:** Uniform rotation process of the outer gimbal for determining the regions with excessive temperature fluctuation of the temperature control system.

Rotation Process	Total Angle (°)	Excessive Fluctuation
360–270°	90	No
Pause	0	No
270–180°	90	Yes
Pause	0	No
180–0°	180	Yes

**Table 2:** Sinusoidal periodic rotation process and relevant parameters of the outer gimbal.

INS Number	Angle Mean Value (°)	Rotational		Temperature	
		Amplitude (°)	Period (s)	Maximum Fluctuation (°C)	Excessive Fluctuation
INS 1#	**64.5**	30	1200	**−0.8125**	Yes
	64.5 + 180			−0.7813 0.5625	
	64.5 + 90			<0.4500	No
	64.5 + 270				
INS 2#	**64.5**			**−0.8125**	Yes
	64.5 + 180			−0.7750 0.6563	
	64.5 + 90			<0.4500	No
	64.5 + 270				

**Table 3:** Results of high–low temperature and heading rotation tests of INS before and after optimization.

Test Method	Subject Tested	Ambient Temperature	Performance Parameters	Test Results	
				Before Optimization (°C)	After Optimization (°C)
High–low temperature test	Gyro 1#	High temperature ^1^	Average bias	0.2404	0.1155
			Maximum bias	0.61	0.54
			Maximum fluctuation difference	**1.4**	**0.5**
		Low temperature ^2^			
			Average bias	1.2187	0.2651
			Maximum bias	1.5	1.0
	Gyro 2#	High temperature	Average bias	0.2039	0.1864
			Maximum bias	0.7	0.5
			Maximum fluctuation difference	**1.5**	**0.5**
		Low temperature			
			Average bias	1.2580	0.4584
			Maximum bias	1.5	1.0
Heading rotation test	Temperature control system	Normal temperature ^3^	Standard deviation	0.2942	0.1096
			Maximum fluctuation	**−0.8125**	**−0.2656**

Notes: ^1^ High temperature: 29 °C, ^2^ Low temperature: 19 °C, ^3^ Normal temperature: 24 °C.
